# Regulation of microRNAs by epigenetics and their interplay involved in cancer

**DOI:** 10.1186/1756-9966-32-96

**Published:** 2013-11-22

**Authors:** Xiaolan Liu, Xiaoyan Chen, Xinfang Yu, Yongguang Tao, Ann M Bode, Zigang Dong, Ya Cao

**Affiliations:** 1 Cancer Research Institute, Central South University, Changsha, Hunan 410078, China; 2 Key Laboratory of Carcinogenesis and Cancer Invasion, Ministry of Education, Changsha, Hunan 410078, China; 3 Key Laboratory of Carcinogenesis, Ministry of Health, Changsha, Hunan 410078, China; 4 Molecular Imaging Center, Central South University, Changsha, Hunan 410078, China; 5 The Hormel Institute, University of Minnesota, 801 16th Avenue NE, Austin, MN 55912, USA

**Keywords:** microRNA, Epigenetic, DNA methylation, Histone modification, Cancer

## Abstract

Similar to protein-coding genes, miRNAs are also susceptible to epigenetic modulation. Although numerous miRNAs have been shown to be affected by DNA methylation, the regulatory mechanism of histone modification on miRNA is not adequately understood. EZH2 and HDACs were recently identified as critical histone modifiers of deregulated miRNAs in cancer and can be recruited to a miRNA promoter by transcription factors such as MYC. Because miRNAs can modulate epigenetic architecture and can be regulated by epigenetic alteration, they could reasonably play an important role in mediating the crosstalk between epigenetic regulators. The complicated network between miRNAs and epigenetic machineries underlies the epigenetic–miRNA regulatory pathway, which is important in monitoring gene expression profiles. Regulation of miRNAs by inducing epigenetic changes reveals promising avenues for the design of innovative strategies in the fight against human cancer.

## Introduction

MicroRNAs (miRNAs) are endogenous non-coding RNAs (~22 nucleotides) that regulate gene expression at the post-transcriptional level. Similar to protein-coding genes, the expression of miRNAs is also regulated by both genetic and epigenetic mechanisms. Approximately half of the miRNA genes are located in fragile regions of the genome that are associated with deletion, duplication or translocation. This suggests that alterations in miRNA genes could be a more general defect in tumor cells [1]. With the recent discovery of epigenetic processes, an increasing number of miRNAs have been discovered to be affected by epigenetic aberrations in tumor cells [2]. Clearly, miRNA genes can be epigenetically regulated by DNA methylation and/or histone modifications. In turn, a subgroup of miRNAs, named epi-miRNAs, was recognized to directly target enzymatic effectors involved in epigenetic modulation [3]. These observations suggest the existence of a regulatory circuit between epigenetic modulation and miRNAs, which could have a significant effect on transcription [4]. Because miRNAs have a large impact on carcinogenesis through the regulation of diverse target genes, understanding the regulatory mechanisms of miRNA expression is important in treatment and prevention of human cancers.

Epigenetic changes such as DNA methylation and histone modification are associated with chromatin remodeling and regulation of gene expression in mammalian development and human diseases, including cancer. The first evidence for the epigenetic regulation of miRNAs in cancer was obtained by using chromatin modifying drugs to reactivate miRNAs at the transcriptional level [5]. Emerging evidence shows that more than one hundred miRNAs are regulated by epigenetic mechanisms, and about one-half of them are modulated by DNA methylation [6]. Because CpG methylation can be analyzed by a variety of techniques with relatively high sensitivity, we can identify miRNAs deregulated by aberrant DNA methylation in primary samples that might be limited in number and of poor quality [7]. However, DNA methylation does not always take place alone, but often occurs in the presence of other epigenetic modifications, such as histone modification, which constitutes the second major epigenetic regulatory system of miRNAs. While DNA methylation leads to miRNA silencing, histone modification, especially histone methylation, can either trigger or suppress miRNA expression, depending on the target amino acid residues and the extent of methylation. Given that miRNA expression is tissue-specific and depends on cellular context, histone modification might regulate distinct subpopulations of miRNAs in different types of cancers. In addition, the analysis of chromatin modification status should be performed on pure cell populations. Accordingly, identifying the specific miRNAs, which are regulated by aberrant histone modification in clinical tissue samples, remains challenging [8]. For the above reasons, the role of histone modification in miRNA deregulation is still obscure and has been poorly elucidated thus far.

In order to systematically understand the regulatory mechanism of histone modification on miRNA expression, in this review we will focus on the current knowledge regarding the miRNAs deregulated by epigenetic mechanisms, especially histone modification, in human cancer. We will also connect the indirect crosstalk between epigenetic regulators through miRNA mediation.

## Epigenetic mechanisms of miRNA dysregulation in cancer

With the progress in DNA methylation detection techniques, numerous miRNAs have been identified that are modulated by DNA methylation, shedding light on the epigenetically regulated miRNAs. Among them, miR-9, miR-148, miR-124, miR-137, miR-34, miR-127 and miR-512 reportedly can be silenced by CpG hypermethylation in at least three types of cancers [6]. However, it is still largely unknown which miRNAs can be altered owing to histone modifications. To date, histone methylation and histone deacetylation were confirmed to be involved in miRNA regulation. Understanding which and how miRNAs are regulated by histone modifying effectors in cancer might be helpful in tumor treatment.

### MiR-29

The miR-29 family, which targets DNA methyltransferase 3 (DNMT3), is the first reported epi-miRNA, and is also the most extensively studied miRNA that is regulated by histone modification [9]. Recent studies show that transcription factors can regulate miRNA expression through epigenetic mechanisms. For instance, MYC can induce epigenetic regulation of miR-29 repression through histone deacetylation and tri-methylation in B-cell lymphomas (BCL), since it can recruit histone deacetylase 3 (HDAC3) and enhancer of zeste homolog 2 (EZH2) to the miR-29 promoter, forming a MYC/HDAC3/EZH2 co-repressor complex. Without MYC, however, the lack of binding of HDAC3 and EZH2 to the miR-29 promoter results in increased miR-29 expression [10]. Therefore, MYC plays an indispensable role in the epigenetic repression of miR-29 by inducing histone deacetylation and histone tri-methylation. Meanwhile, EZH2 can also repress miR-494 to create a positive feedback loop, which in turn increases MYC abundance and then sustains miR-29 repression in BCL [10]. These properties indicate that different epigenetic modifications can cooperatively regulate the same miRNA, whereas a specific epigenetic effector can regulate more than one miRNAs in the same type of tumor.

Previous research evidence suggested that the transcription factor Yin and yang 1 (YY-1) can recruit various proteins such as EZH2 and HDACs to target genes during various epigenetic events [11-13]. Later Wang et al. confirmed that nuclear factor κB (NF-κB) up-regulated YY-1 resulted in the recruitment of EZH2 and HDAC1 to the miR-29 promoter in myoblasts, leading to the down-regulation of miR-29 and maintaining cells in an undifferentiated state. Once myogenesis starts, the repressive complex containing YY-1/EZH2/HDAC will be replaced by an activating complex. Therefore, miR-29 is restored and in turn targets YY1 to ensure differentiation. However, the constitutively activated NF-κB–YY1 in rhabdomyosarcoma (RMS) could induce epigenetic repression of miR-29 and thereby block differentiation. The inhibition of NF-κB activity by inhibitor of nuclear factor κB α (IκBα) would remarkably decrease the level of YY1, and consequently neither EZH2 nor HDAC1 could be recruited to miR-29 promoter [14]. This study demonstrated that NF-κB might be an upstream regulator of the epigenetic status of miR-29 in skeletal myogenesis.

In addition to these effects in solid tumors, miR-29 deregulation by epigenetic mechanisms can also be found in human hematological cancers. For instance, in acute myeloid leukemia (AML), the transcriptional complex NF-κB/Sp1 can interact with HDAC1 and HDAC3 to form the NF-κB/Sp1/HDAC complex on miR-29b enhancer, which resulted in the silencing of miR-29b. Notably, MYC can directly bind to miR-29b promoter and stimulate the activity of NF-κB/Sp1/HDAC. Therefore, the down-regulation of miR-29b is MYC-dependent [15]. Interestingly, HDAC inhibition could restore the expression of miR-29b in only one third of chronic lymphocytic leukemia (CLL) samples [16]. For the other two-thirds of CLL cases, the identification of other histone modifications that contribute to epigenetic silencing of miR-29b still needs to be accomplished.

In summary, binding of MYC or NF-κB on the miR-29 promoter seems to be a primary event in miR-29 silencing, and thereby induces the initial step of its chromatin modification. Subsequently, various histone modifying enzymes such as EZH2 and HDACs can be recruited to the miR-29b promoter. These enzymatic effectors might receive signals from their initiator, and then function as an executor of this epigenetic event. Additionally, the transcription factors YY1 and Sp1, which are dispensable in this regulation, might act as bridges that connect the initiator and the executor.

### Let-7 family

Reportedly, the let-7 miRNAs, which target oncogenic Ras and function as tumor suppressors, are located in fragile genomic regions that are frequently deleted in human cancers [1,17]. Besides genomic alterations, the let-7 genes could also be regulated by epigenetic mechanisms. MYC induced by *H. pylori* CagA in gastric cancer cells can suppress the expression of let-7a and let-7c through two epigenetic approaches: (1) MYC stimulates EZH2 expression by reducing its negative regulators, miR-26a and miR-101; (2) MYC interacts with DNMT3B and EZH2 on the let-7 promoter, and consequently the let-7 gene is silenced through both DNA and histone methylation. Accordingly, the Ras pathway is activated to contribute to carcinogenesis [18]. However, in human lung cancers, let-7a-3 was found to be hypomethylated, which is different from its status in normal lung tissues [19], suggesting that differential, and even opposite, epigenetic regulations might take place in the same miRNA according to the cell context. In view of that, exploration into the epigenetic modulation of the let-7 gene family is essential.

### MiR-15a/miR-16 cluster

The miR-15a and miR-16 are located on the human chromosome 13q14, and consequently their levels could be lowered by deletions in 13q14, which occur commonly in CLL and mantle cell lymphoma (MCL) [20]. However, down-regulation of these two miRNAs is also observed in many CLL cases with intact chromosome 13 [21], indicating that other mechanisms might be involved in this regulation. Recently, HDAC inhibition was proposed to trigger the expression of miR-15a and miR-16 in some CLL samples, suggesting they could be epigenetically silenced by histone deacetylation [16]. Interestingly, Zhang et al. revealed that MYC repressed miR-15a/16-1 cluster expression through recruitment of HDAC3 in MCL [22], emphasizing that MYC plays an important role also in the epigenetic silencing of the miR-15a/miR-16 cluster.

### MiR-31

Like the miR-15a/miR-16 cluster, miR-31 is also considered to be both genetically and epigenetically regulated. Genetic loss of miR-31, which resides in the deletion hotspot 9p21.3, was demonstrated to be beneficial for tumor progression and was observed in several types of human cancers [23]. However, the loss of miR-31 expression can also be detected in tumor cells without 9p21.3 deletion. DNA methylation and/or EZH2-mediated histone methylation were recently confirmed to contribute to miR-31 loss in melanoma, breast cancer and adult T cell leukemia (ATL) [24-26]. Also ChIP-PCR assay results revealed the YY1 binding motifs around the miR-31 region, which recruit EZH2 and mediate epigenetic silencing of miR-31. Although YY1 could contribute to miR-31 repression, knockdown of YY1 in ATL cells without genetic deletion only restored a small proportion of the silenced miR-31 and could not remove EZH2 completely from the miR-31 region [26]. Thus, YY1 does not appear to be indispensable in EZH2-mediated miR-31 silencing, pointing out the existence of other important upstream regulators.

### MiR-23a

MiR-23a was demonstrated to be transcriptionally repressed by MYC in many cancer cells [27]. Besides MYC, other transcription factors can also epigenetically regulate miR-23a expression. For instance, the NF-κB p65 subunit can recruit HDAC4 to miR-23a promoter, thereby silencing the expression of miR-23a in human leukemic Jurkat cells [28]. HDAC4 as a member of class IIa HDACs is expressed tissue-specifically in heart, smooth muscle and brain [29]. Thus, compared with the widely expressed class I HDAC enzymes (HDAC1, -2, -3, and -8), HDAC4 seems to have a tissue-restricted role in epigenetic regulation of miRNAs.

### Other down-regulated miRNAs

In addition to the above miRNAs, multiple miRNAs that are downregulated by histone modifications also exist. For instance, miR-139-5p, miR-125b, miR-101, let-7c, miR-200b were found to be epigenetically repressed by EZH2, and miR-449 was repressed by HDACs in human hepatocellular carcinoma (HCC) [30,31]. Similarly, EZH2 suppressed the expression of miR-181a, miR-181b, miR-200b, miR-200c, let-7 and miR-203 in prostate cancer [32,33]. Moreover, the histone demethylase Jarid1b could also repress let-7e as well as miR-1246, miR-1826, and miR-361-5p by removing the active mark H3K4me3 in breast cancer [34]. However, the underlying molecular mechanisms of these miRNAs are still unknown and should be studied in detail.

### Up-regulated miRNAs

Most of the miRNAs deregulated by aberrant patterns of histone modification in cancer cells are silenced, but some miRNAs, such as miR-224, miR-615 and miR-155, are activated by histone modification.

The miR-224 is the most significantly upregulated miRNA in HCC and was found to target apoptosis inhibitor-5 (API-5) to promote tumorigenesis [35]. However, the regulatory mechanism of miR-224 in liver disease is mostly obscure. Actually, miR-224 overexpression can be attributed to histone acetylation rather than genomic amplification or DNA hypomethylation. The histone acetylase protein EP300 acts as a positive regulator in this regulation, whereas HDACs function as negative regulators [36]. Considering that miR-224 overexpression could not be totally attenuated by inhibition of histone acetylation, other factors might also contribute to miR-224 upregulation. Similarly, a study in prostate cancer cells identified miR-615 as an epigenetically activated miRNA by DNA methylation loss and H3K9 acetylation gain [37].

As an oncogenic miRNA, miR-155 is overexpressed in many cancers such as breast cancer [38,39]. Recently, miR-155 in normal breast tissues was proposed to be epigenetically repressed by wild-type BRCA1, which interacted with HDAC2 to deacetylate H2A and H3 on the miR-155 promoter. In BRCA1-deficient or BRCA1-mutant cancer cells, however, the loss or mutation of BRCA1 resulted in miR-155 upregulation, since HDAC2 could not be recruited to the miR-155 promoter [40].

**Figure 1:**
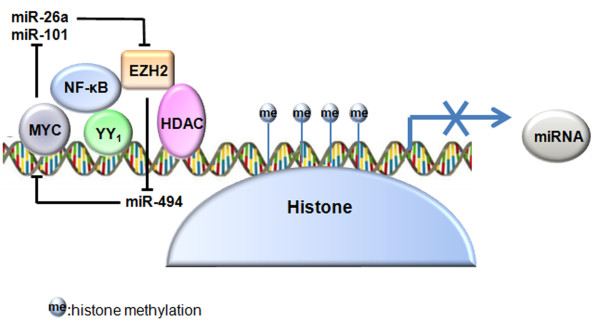
**A model depicting the mechanisms of histone modification that repress miRNA expression.** MYC or NF-κB, which interacts with transcription factor YY1 or Sp1 on miRNA promoter, is hypothesized to be the upstream regulator of miRNA silencing. Various histone modifying enzymes such as EZH2 and HDACs can be recruited to methylate and deacetylate histones. A positive feedback loop exists between MYC and EZH2: MYC stimulates EZH2 expression by reducing its negative regulators, miR-26a and miR-101; EZH2 can also increase the abundance of MYC by repressing miR-494.

The regulatory models of miR-29 and other miRNAs suggest that the well-known transcription factor MYC, which is one of the most commonly overexpressed oncogenes in cancer, has some functions in the aspect of epigenetic regulation (Figure 1).

## The crosstalk between epigenetic regulators

**Figure 2:**
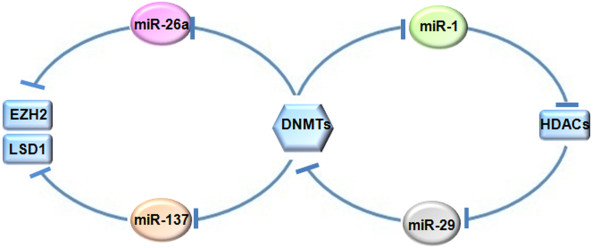
**The role of miRNAs in mediating the crosstalk between epigenetic regulators.** DNMT1 contributes to miR-1 silencing in HCC cells, thereby promoting the accumulation of its target HDAC4. The miR-29, which targets DNMT3, is down-regulated by HDACs in AML. Likewise, miR-26a and miR-137 are silenced by promoter CpG island hypermethylation, which induces the up-regulation of the target gene LSD1 in colorectal adenomas and EZH2 in prostate cancer.

The importance of inhibitory signals that contribute to epigenetic gene silencing, especially DNA methylation and histone deacetylation, has been increasingly recognized in recent years. However, the crosstalk between these epigenetic regulators is not fully understood, because of the difficulty to apply a unique model that can explain DNA and histone modification in specific epigenetic events. Nevertheless, accumulating data support the existence of a circulation loop between DNA methylation and histone modification, i.e., DNA methylation directs histone modification and histone modification recruits more DNA methylation. All of these observations suggest a reciprocal crosstalk between DNA methylation and histone modification. Indeed, these epigenetic regulators can communicate and benefit each other to reinforce epigenetic gene silencing. In this scenario, miRNAs are becoming a crucial factor in the faithful transmission of different patterns of epigenetic modulation (Figure 2).

The miR-26a can be silenced by DNMTs in prostate cancer, which induces the accumulation of its target gene EZH2 and changes the global DNA methylation status [41], supporting the idea that miRNAs can mediate the interplay between epigenetic regulators. The miR-137 is another important mediator, which is silenced by promoter CpG island hypermethylation and targets lysine-specific demethylase 1 (LSD1) in colorectal adenomas [42]. Because LSD1 can stabilize DNMT1, a positive feedback loop exists between them. Besides the crosstalk between DNA and histone methylation, indirect crosstalk between DNA methylation and histone deacetylation also occur through miRNA mediation, such as miR-1 and miR-29. The miR-1, which targets HDAC4, is down-regulated in human HCC cells because of its CGI hypermethylation by DNMT1, thereby promoting the expression of HDAC4 [43]. Likewise, HDACs can induce miR-29 silencing in acute myeloid leukemia (AML), which in turn increases the expression of its target gene DNMT3 [15,44].

These findings indicate that epigenetic information can flow from one modulation to a miRNA, and then from the miRNA to another epigenetic pattern. As a member of epigenetic machinery, miRNAs can also contribute to the conversation between other epigenetic events.

## Controlling miRNA expression with epigenetic drugs

The frequent dysregulation of miRNAs and their interplay with epigenetic regulators in cancer make them attractive biomarkers and prospective therapeutic targets in clinical applications. The therapeutic application of miRNAs in cancer involves two strategies: 1) inhibition of oncogenic miRNAs by using miRNA antagonists, such as anti-miRs or antagomiRs; or 2) introduction of tumor suppressor miRNAs through either synthetic miRNA mimics or by stable and vector-based transfection of genes coding for miRNAs [45]. Moreover, a deeper understanding of these epigenetically regulated miRNAs might lead to a novel therapeutic strategy based on using of epigenetic drugs to control the expression of both onco-miRNAs and tumor-suppressor-miRNAs for prevention or treatment of human cancers.

The abnormalities of epigenetic in cancer, unlike genetic lesions, can be reversed by epigenetic-regulated drugs, which provides an opportunity for epigenetic therapy. The goal of epigenetic therapy would be to target the chromatin in rapidly dividing tumor cells in order to bring them to a more 'normal state’, while only mildly disturbing the epigenome of healthy cells [46]. Five kinds of epigenetic drugs are known, including DNMT inhibitors, HDAC inhibitors, histone acetyltransferase (HAT) inhibitors, histone methyltransferase (HMT) inhibitors and histone demethylase (HDT) inhibitors [47]. Most of the research efforts focused on the first two agent types. For example, two DNMT inhibitors, 5-azacytidine (5-AzaC) and 5-aza-2′-deoxycytidine (5-Aza-CdR), were approved by FDA to treat myelodysplastic syndromes (MDS) and AML [48]. In 2006, the FDA first approved the HDAC inhibitor suberoylanilide hydroxamic acid (SAHA) to treat cutaneous T-cell lymphoma (CTCL) [49]. Probably, with the discovery and elucidation of epigenetic–miRNA regulatory pathways, at least part of the observed therapeutic effects of these epigenetic agents, such as 5-Aza-CdR, might be attributed to their effect on miRNAs.

The deregulated miRNAs that can be controlled by epigenetic drugs in human cancers are shown in Table 1. These agents can either cause the re-expression of silenced tumor suppressor miRNAs or repress oncogenic miRNAs that are over- expressed in cancer cells. Besides the most commonly used DNMT inhibitors and HDAC inhibitors, C646 is a novel HAT inhibitor that is able to inhibit histone acetyltransferase EP300 and suppress the upregulated miR-224 [36]. However, these drugs might work better together than individually. For example, the combined use of 3-deazaneplanocin A (DZNep) and trichostatin A (TSA), but not their single use, could dramatically induce miR-449 expression [50]. One possible reason for this activity is that miRNA genes are regulated by multiple epigenetic effectors, and thus inhibition of one factor might not reverse miRNA expression completely. Consequently, the idea of combining different types of epigenetic drugs to effectively control abnormal miRNA expression in cancer cells turns out to be quite exciting and attractive.

**Table 1:** The deregulated miRNAs that can be controlled by epigenetic drugs in human cancers

**Epigenetically regulated miRNAs**	**Target protein**	**Cancer type**	**Epigenetic drug**	**Fold change**	**Citation**
***Down-regulated***					
miR-449	CDK6, CDC25A, c-MET	Breast cancer	DZNep + TSA	>20 folds	[50]
		Hepatocellular carcinoma	TSA	10-30 folds	[31]
miR-125b	PIGF	Hepatocellular carcinoma	5-Aza-CdR	>2 folds	[51]
miR-126	VEGFA, PIK3R2	Bladder cancer	5-Aza-CdR + PBA	>10 folds	[52]
miR-127	BCL6	Bladder cancer and clear cell renal cell carcinomas	5-Aza-CdR + PBA/TSA	>10 folds	[5,53]
miR-1-1	FOXP1, MET, HDAC4	Hepatocellular carcinoma	5-AzaC	>20 folds	[43]
miR-34	C-MYC, CDK6,	Lung cancer, melanomas and prostate cancer	5-Aza-CdR	>10 folds	[54,55]
miR-181a,b, miR-200a,b,c, and miR-203	BMI1 and RING2	Prostate cancer	DZNep/5-Aza-CdR/SAHA	2-20 folds	[32]
miR-31	SRC, RAB27a, NIK and MET, RhoA and WAVE3	Melanoma	5-Aza-CdR/DZNep	>2 folds	[24]
		Breast cancer	5-Aza-CdR or 5-Aza-CdR + TSA	>5 folds	[25]
miR-29	Mcl-1, DNMT3A, DNMT3B,SP1, Tcl-1, CDK6 and IGR1F	Aggressive B-Cell Lymphomas	DZNep + SAHA	2-10 folds	[10]
		Chronic lymphocytic leukemia and acute myeloid leukemia	LBH589/SAHA/AR-42	2-5 fold	[15,16]
miR-15a/miR-16	BCL-2 and MCL-1	Chronic lymphocytic leukemia	LBH589/SAHA	2-5 fold	[16]
miR-205	SIP1 and ZEP	Prostate cancer	5-Aza-CdR	>100 folds	[37]
miR-21	PDCD4, TPM1, and MARCKS	Prostate cancer	5-Aza-CdR	>2 folds	[37]
miR-370	IRS-1	Oral squamous cell carcinoma	5-Aza-CdR	2-20 folds	[56]
miR-373	MBD2	Hilar cholangiocarcinoma	5-Aza-CdR or 5-Aza-CdR + TSA	2-5 folds	[57]
miR-193b	ETS1, CCND1, PLAU	Prostate cancer	5-Aza-CdR + TSA	>2 folds	[58]
miR-132	HB-EGF,TALIN2	Prostate cancer	5-Aza-CdR or 5-Aza-CdR + TSA	>5 folds	[59]
miR-145	BNIP3, TNFSF10,PAK7	Prostate cancer and clear cell renal cell carcinomas	5-Aza-CdR or 5-Aza-CdR + TSA	>2 folds	[53,60]
miR-141, miR-514	HGF, CDC42, RBX1, SLC2A1	Clear cell renal cell carcinomas	5-Aza-CdR + TSA	>2 folds	[53]
miR-335	SOX4, Rb1	Hepatocellular carcinoma	5-Aza-CdR + TSA	>2 folds	[61]
miR-205	BCL-w	Prostate cancer	5-Aza-CdR	2-5 folds	[62]
let-7 family	RAS	Nasopharyngeal carcinoma	Zebularine	2-100 folds	[63]
***Up- regulated***					
miR-224	API-5	Hepatocellular carcinoma	C646	<50%	[36]
miR-17-92 Cluster	PTEN, BCL2L11, CDKN1A	Colorectal Cancer	SAHA or TSA	25%-67%	[64]

DNMT inhibitors: 5-azacytidine (5-AzaC); 5-aza-2′-deoxycytidine(5-Aza-CdR); Zebularine.

HDAC inhibitors: suberoylanilide hydroxamic acid (SAHA); trichostatin A (TSA); panobinostat(LBH589); phenylbutyrate (PBA); OSU-HDAC42(AR-42).

HAT inhibitors: C646.

HMT inhibitors: 3-deazaneplanocin A (DZNep).

## Conclusion

The connection between DNA methylation and histone modification has been described in several physiological systems and disease states. Recent investigations also added miRNAs to this complicated crosstalk [65], thereby reinforcing the effects of epigenetic reprogramming. A better understanding of epigenetic regulatory mechanism of miRNA expression will help to elucidate the complex network of epigenetic modifications and design innovative strategies for cancer treatment. Although DNA methylation is the best-studied epigenetic mechanism for miRNA deregulation, it is still largely unknown which miRNAs are altered owning to histone modification. This is partially due to the lack of effective detection methods and relatively strict requirements for obtaining and examining clinical samples.

Notably, the expanding role of MYC in epigenetics has recently received further attention. First, MYC can promote the expression of epigenetic enzymes, which contribute to tumor development. For example, MYC can induce the accumulation of EZH2 in prostate cancer [66]. Second, recent evidence attributed the deregulated miRNA expression to MYC, which is involved in promoting oncogenic miRNAs and repressing tumor suppressor miRNAs [67,68]. Considering the known mechanisms of histone modification, MYC might function as an initiator of miRNA epigenetic silencing, which can recruit enzymatic effectors such as HDAC and EZH2 to the miRNA promoter. Conversely, HDT and HAT are rarely reported in miRNA regulation, pointing out the needing to evaluate the potential of epigenetic drugs to re-express or repress deregulated miRNAs that contribute to carcinogenesis. Owing to the reversible nature of epigenetic alterations, therapeutic strategies targeting specific miRNAs based on epigenetic intervention might provide innovative tools for cancer treatment in the future. Further understanding of epigenetic mechanisms in miRNA regulation along with the effect of epigenetic drugs on specific miRNAs might help to reset the abnormal cancer epigenome.

## Abbreviations

DNMTs: DNA methyltransferases; BCL: B-cell lymphomas; HDACs: Histone deacetylases; EZH2: Enhancer of zeste homolog 2; YY-1: Yin and yang 1; NF-κB: Nuclear factor κB; RMS: Rhabdomyosarcoma; IκBα: Inhibitor of nuclear factor κB α; CLL: Chronic lymphocytic leukemia; MCL: Mantle cell lymphoma; ATL: Adult T cell leukemia; API-5: Apoptosis inhibitor-5; HCC: Hepatocellular carcinoma; TSA: Trichostatin A; AML: Acute myeloid leukemia; HATs: Histone acetyltransferases; HMTs: Histone methyltransferases; HDTs: Histone demethylases; LSD1: Lysine-specific demethylase 1; 5-AzaC: 5-azacytidine; 5-Aza-CdR: 5-aza-2′- deoxycytidine; MDS: Myelodysplastic syndromes; SAHA: Suberoylanilide hydroxamic acid; CTCL: Cutaneous T-cell lymphoma; LBH589: Panobinostat; PBA: Phenylbutyrate; AR-42: OSU-HDAC42; DZNep: 3-deazaneplanocin A.

## Competing interests

The authors declare that they have no competing interests.

## Authors’ contributions

XLL and YC were the main authors of the manuscript; XYC and XFY contributed to bibliography collection as well as figures and tables design and format; YGT revised the manuscript for important intellectual content; AB corrected the language form; ZGD was responsible for the manuscript writing and sequence alignment. All authors read and approved the final manuscript.
